# Complete mitochondrial genome sequence of *Aequorea coerulescens*

**DOI:** 10.1080/23802359.2019.1660257

**Published:** 2019-09-03

**Authors:** Jianing Lin, Song Feng, Lijuan Wang

**Affiliations:** a Department of Riverine Ecological Conservation and Restoration, Chinese Research Academy of Environmental Sciences, Beijing, China;; b CAS Key Laboratory of Marine Ecology and Environmental Sciences, Institute of Oceanology, Chinese Academy of Sciences, Qingdao, China;; c Laboratory for Marine Ecology and Environmental Science, Qingdao National Laboratory for Marine Science and Technology, Qingdao, China;; d Center for Ocean Mega-Science, Chinese Academy of Sciences, Qingdao, China

**Keywords:** *Aequorea coerulescens*, giant jellyfish, hydromedusa, complete mitochondrial genome, Hydra spp

## Abstract

The complete mitochondrial genome sequences of hydromedusa *Aequorea coerulescens*, a dominant giant jellyfish distributing in the Yellow Sea and northern East China Sea, China, is first reported in this research. Its mitochondrial DNA has 14,804 bp in length with a linear structure, containing 13 protein-coding genes (PCGs), 2rRNA (s-rRNA and l-rRNA), and 2tRNA (trnaW-TGA and tranM-AGT). The A + T content of the whole base composition of the genome is 72.8% (A: 30.15%; C: 12.33%; G: 14.87%; T: 42.64%). ATG, ATA, and ATT are start codons in five (ATP8, ATP6, COX3, NAD6, NAD4l), four (NAD2, NAD5, NAD1, COX1) and two PCGs (NAD3, NAD4), respectively. COB and COX2 began with GTG and CAA as start codon, respectively. TAA and ATA were the stop codon of ATP6 and NAD5 as well as NAD2 and NAD4, respectively. However, other PCGs were terminated with different stop codons. The NJ phylogenetic tree among the related 15 jellyfish species showed that *A. coerulescens* is close to *Hydra* spp.

Jellyfish is attracting more and more attention such as *Aurelia* spp., *Nemopilema nomurai*, *Aequorea* spp. etc., because their frequent blooms around the globe have posed severe threats on the human economic development, social security, and marine ecological health in recent decades (Purcell et al. 2007; Purcell 2018). However, the molecular phylogenetic relationship among jellyfish has been rarely investigated by mitochondrial genomes. Among more than 1400 identified jellyfish species (Arai 1997; Mianzan 1999; Pugh 1999; Bouillon and Boero 2000), studies on mitochondrial genomes were reported in only a dozen of species up to date, such as *Aurelia* spp. (Shao et al. 2006; Hwang, Park, Won, Lee et al. 2014), *Craspedacusta sowerbyi* (Zou et al. 2012), *Chrysaora quinquecirrha* (Hwang, Park, Won, Lee, Shin et al. 2014), *N. nomurai* and *Rhopilema esculentum* (Wang and Sun 2017a, 2017b).

*Aequorea coerulescens* belongs to Leptomedusae, Conica, Aequoreae, which was a predominant giant jellyfish in the Yellow Sea and northern East China Sea, China, and massively appeared from April to June (Wang et al. 2012; Zhang et al. 2012). In order to comprehend the molecular phylogenetic relationships between *A. coerulescens* and other jellyfish better, we studied its complete mitochondrial DNA and obtained the basic genetic information of *A. coerulescens* population in Yellow Sea and northern East China Sea, China. Medusae of *A. coerulescens* were collected from the central Yellow Sea (34.5°N, 124°E) and then fixed with 95% ethanol in a − 40 °C refrigerator. Finally, they were stored at an aquarium in the Institute of Oceanology, Chinese Academy of Sciences. The bell tissue of medusae was used for the processing of mitochondrial DNA.

The complete mitochondrial genome of *A. coerulescens* was a linear molecule with 14,804 bp in length (GenBank accession No. MN066550). It contains 13 protein-coding genes (PCGs), small and large subunit ribosomal RNAs (s-rRNA and l-rRNA), methionine and tryptophan transfer RNAs (trnaW-TGA and tranM-AGT). The overall base composition of mitochondrial genome was 30.15% for A, 42.64% for T, 14.87% for G and 12.33% for C, respectively. The A + T base composition (72.8%) was over 70% consistent with *Hydra* spp. (Kayal and Lavrov 2008; Pan et al. 2014).

For 13 protein-coding genes (PCGs), there are 5 PCGs started with ATG codon (ATP8, ATP6, COX3, NAD6, NAD4l), 4 with ATA codon (NAD2, NAD5, NAD1, COX1), 2 with ATT (NAD3, NAD4), 1 with GTG (COB), and 1 with CAA (COX2). All the genes appeared complete stop codons. However, there are only 2 PCGs terminated with TTA codon (ATP5 and NAD5) and 2 with ATA (NAD2 and NAD4). Other PCGs end with different stop codons (GAA for COX2, AGC for ATP8, GGA for COX3, GGT for NAD6, TGA for NAD3, AAA for NAD4L, CCT for NADl, GTA for COB, TCA for COX1). The very slight anti-G bias was found on the 3rd position of PCGs (6.92%). The NJ phylogenetic tree among 15 species was formed based on the complete mitochondrial genome from NCBI (Figure 1). The results found that *A. coerulescens* is close to *Hydra* spp. (GenBank No. NC021406.1 and NC010214.1).

**Figure 1.**
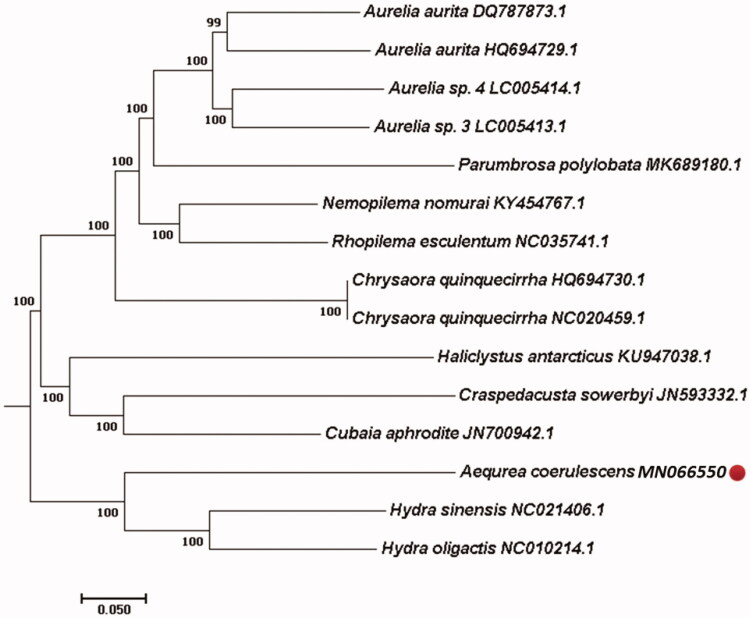
Phylogenetic relationship revealed by NJ tree.
